# Cell Type Diversity in Hepatitis B Virus RNA Splicing and Its Regulation

**DOI:** 10.3389/fmicb.2019.00207

**Published:** 2019-02-08

**Authors:** Noriomi Ito, Kenji Nakashima, Suofeng Sun, Masahiko Ito, Tetsuro Suzuki

**Affiliations:** Department of Virology and Parasitology, Hamamatsu University School of Medicine, Shizuoka, Japan

**Keywords:** hepatitis B virus, alternative splicing, *cis*-acting element, intronic silencer, exonic enhancer

## Abstract

Although RNA splicing of hepatitis B virus (HBV) is a commonly observed in livers of hepatitis B patients as well as in the cultured cells replicating the viral genome, its biological significance in the HBV life cycle and the detailed regulatory mechanisms are still largely unclear. In this study, we found cell-type dependency of HBV splicing of the 3.5 kb pregenomic RNA, which is efficiently spliced in human hepatoma cells but not in cells derived from human hepatic stellate, mouse hepatoma and human non-hepatic cells. It may be likely that RNA splicing is one of the determinants of host range restriction of HBV. Given the finding indicating the difference in cell-type dependency of the splicing efficiency between HBV and simian virus 40, we carried out intron-swapping experiments. The results suggest the presence of putative exonic splicing enhancer that possibly works in the cell-type dependent fashion. Together with further mutational analyses, a novel 50-nt intronic splicing silencer, whose secondary structure is well conserved among the HBV strains, was identified. It appears that this intronic silencer functions effectively independent of cell backgrounds.

## Introduction

Hepatitis B virus (HBV) infection is a major global problem despite the availability of an efficacious vaccine. In chronic HBV infection, liver cirrhosis and hepatocellular carcinoma (HCC) are associated with considerable morbidity and mortality. Approximately 240 million people worldwide are chronically infected with the virus (El-Serag, 2012). HBV is a member of the *Hepadnaviridae* family and contains a 3.2 kb partially double-stranded relaxed circular DNA genome with four open reading frames encoding seven proteins.

Upon infection, the uncoated viral genome is transported to the nucleus and converted into covalently closed circular DNA, which is a stable form of the viral genome and serves as the template for synthesis of viral transcripts. Four unspliced viral RNAs, 3.5, 2.4, 2.1, and 0.7 kb, are transcribed from their respective promoters and two enhancer regions and end at common polyadenylation signal located in the core open reading frame. The 3.5 kb RNA includes precore and pregenomic RNA species. Precore mRNA codes for precore antigen or HBeAg. The pregenomic RNA serves as a template for the synthesis of HBV DNA and also as the mRNA of core antigen (HBcAg) and polymerase. In addition, the 3.5 kb RNA can be alternatively spliced to generate at least 14 splice variants that have been identified in sera and livers of hepatitis B patients (Candotti and Allain, 2017). In cultured human hepatoma cells transfected with the viral genome, synthesis of multiple spliced RNAs derived from 3.5 kb RNA has been commonly observed among HBV isolates. It has been reported that up to 80% of intracellular capsids contain the viral DNAs originated from the spliced RNAs in HBV genome-replicating hepatoma cells (Terre et al., 1991; Rosmorduc et al., 1995; Soussan et al., 2008; Redelsperger et al., 2012; Bayliss et al., 2013). The major spliced variant termed as SP1, which has an intron between nt 2448 and 488, may account for up to 30% of total 3.5 kb RNA (Gunther et al., 1997; Sommer et al., 2000; Duriez et al., 2017).

RNA splicing is an essential step for eukaryotic gene expression and is tightly regulated in different tissues and developmental stages. While this process depends on recognition of short well-conserved splice site sequences at the exon–intron boundaries, additional *cis*-acting splicing regulatory elements, which act as either enhancers or silencers of splicing and are potentially present either in exons or introns, play key roles in defining specificity and efficiency of splicing. In terms of splicing regulatory elements of HBV, the post-transcriptional regulatory element (PRE), a highly structured *cis*-acting sequence of approximately 0.5 kb, was identified within the viral transcripts. PRE is located near the 3′ end region of all HBV mRNAs and is known to be involved in regulation of the 3.5 kb RNA splicing (Heise et al., 2006). Heterologous minigene contexts were used for investigating *cis*-acting regulatory elements and distinct sequences that can function as positive and negative regulators for the 3.5 kb RNA splicing were identified within the PRE (Huang et al., 2011). HBV intronic *cis*-element was also examined and an intronic splicing silencer-long, ISS_L_, was identified (Chowdhury et al., 2011). However, biological significance of HBV RNA splicing, especially in terms of viral replication and/or host range as well as the detailed regulatory mechanisms underlying post-transcriptional processing events in the HBV life cycle are still largely unclear.

In this study, we compared expression of HBV 3.5 kb RNA and spliced form(s) derived from the RNA in 13 kinds of cell lines transfected with the viral genome and found that the 3.5 kb RNA can be relatively efficiently spliced in human HCC cells compared to other types of cells. Findings obtained from intron-swapping experiments between HBV and simian virus 40 (SV40) suggest involvement of a putative exonic enhancer in regulation of alternative splicing in a cell-type dependent manner. Further, we identified a novel intronic silencer element spanning at nt 2877–2926 that was well conserved in the predicted structural conformation among HBV strains.

## Materials and Methods

### Plasmids

Plasmids containing the 1.24-fold HBV genomes derived from HBV genotypes (GTs) A (NCBI No. AB246337), B (NCBI No. AB246342) and D (NCBI No. AB246347); pUC-HBV-Ae, -Bj56 and -D_Ind60, respectively, were gifts from Dr. Mizokami of National Center for Global Health and Medicine, Japan (Sugauchi et al., 2004a,b). pUC-HBV-Ce was 1.24-fold HBV genome derived from a consensus sequence of HBV GT-C (subgenotype Ce), as described (Sun et al., 2017). A series of deletion mutants with or without substitutions in the HBV sequence corresponding to major intron of the splicing, as indicated in Figure 3A, were generated based on pUC HBV-Ce, -Ae and -D_Ind60, which were used as templates for PCR. pCAG-SV40 that expresses SV40 late gene under the CAG promoter (Nakanishi et al., 2008) was provided by Dr. Nakanishi of National Institute for Longevity Sciences, Japan. HBV-based chimeric plasmid carrying the SV40 intron region, pHBV/int-SV40, was constructed as follows. HBV cDNA corresponding to the major splicing intron region (1,256 bp) in pUC HBV-Ce was deleted by PCR and used for cloning the 932 bp of SV40 cDNA fragment of the splicing intron region that was amplified by PCR using pCAG-SV40 as a template, by using an In-Fusion HD cloning kit (Takara Bio, Shiga, Japan). In the same way, to create SV40-based chimeric plasmid carrying the HBV intron region, pSV40/int-HBV, a 1,256 bp fragment of HBV cDNA of the splicing intron was amplified, followed by cloning the fragment into the pCAG-SV40-based linearized vector in which the sequence of the SV40 splicing intron was deleted. A series of mutants pUC HBV-Ce-D1, -D2, -D3, -D4, -D5, -S1/D1, -S1/D1/S2 and -S1, which carry partial deletions and/or substitutions within the region corresponding to the major intron, were generated via several PCRs using pUC HBV-Ce as the template, followed by self-ligation of the synthesized fragments. Nucleotide sequences of the regions introducing substitution mutations S1 and S2 (Figure 3A) in pUC HBV-Ce-S1/D1, -S1/D1/S2 and –S1 were shown (Supplementary Table S1). To generate a series of HBV GT-C-based chimeric plasmids harboring parts of GT-A sequences (HBV Ce/Ae1-Ce/Ae7), various parts in the HBV genome were removed from pUC-HBV-Ce, followed by replacing with corresponding parts from pUC-HBV-Ae. All mutated or chimeric constructs generated were sequenced to ensure that no mutations were introduced.

### Cell Culture and Transfection

Human HCC cells; HuH-7, HepG2, PLC/PRF5, FLC7 (Homma et al., 1990) and ORL8c (Kato et al., 2009), human hepatic stellate TWNT4 JP7 cell (Shibata et al., 2003; Chida et al., 2017), mouse hepatoma Hepa1c1c7 cell and human non-hepatic cells; A549 (lung carcinoma), HEK293 (embryonic kidney), HEK293T (embryonic kidney), Caco2 (colorectal adenocarcinoma), C33A (cervical carcinoma), and HeLa (cervical adenocarcinoma) were maintained in Dulbecco’s modified Eagle medium supplemented with 10% fetal bovine serum. Cells were transiently transfected with plasmid DNAs using transfection reagents as follows; Lipofectamine LTX (Thermo Fisher Scientific, Waltham, MA, United States) used for HuH-7, FLC7, A549, Caco2, HeLa, Hepa1c1c7, and TWNT4 JP7, Lipofectamine 3000 (Thermo Fisher Scientific) for HepG2, PLC/PRF/5, ORL8c, and C33A, and linear polyethyleneimine, MW 25,000 (Thermo Fisher Scientific) for HEK293 and HEK293T.

### Northern Blot and Western Blot Analyses

Northern blotting was performed as previously described (Sun et al., 2017). Briefly, total RNA samples extracted from cells transfected with HBV plasmids were separated on 1.2% agarose gel with 7% formaldehyde. The samples were transferred to a nylon membrane (Roche Diagnostics, Basel, Switzerland), followed by cross-linking to the membrane by ultraviolet light. The blot was prehybridized with DIG Easy Hybridization buffer (Roche Diagnostics) and hybridized with an HBV RNA probe (Supplementary Figure S1A) labeled with DIG-11-UTP using the DIG Northern Starter Kit (Roche Diagnostics). To generate a DIG-labeled RNA probe that specifically binds to HBV 3.5 kb RNA and its spliced forms, a PCR fragment covering the nt 1998–2447 region was used as templates for *in vitro* transcription. The signals were detected with CDP-Star reagent (GE Healthcare, Buckinghamshire, United Kingdom). Western blotting was performed as previously described (Li et al., 2016). Briefly, the proteins in cell lysates were separated by SDS-PAGE and transferred onto polyvinylidene difluoride membranes. After blocking, membranes were probed with primary antibodies, followed by incubation with peroxidase-conjugated secondary antibody. Antigen-antibody complexes were visualized using ECL Prime Western Blotting Detection Reagent (GE Healthcare).

### Determination of Quantity Ratio of the Spliced RNA to Unspliced 3.5 kb RNA or Total 3.5 kb RNA Derived Species

**FIGURE 1:**
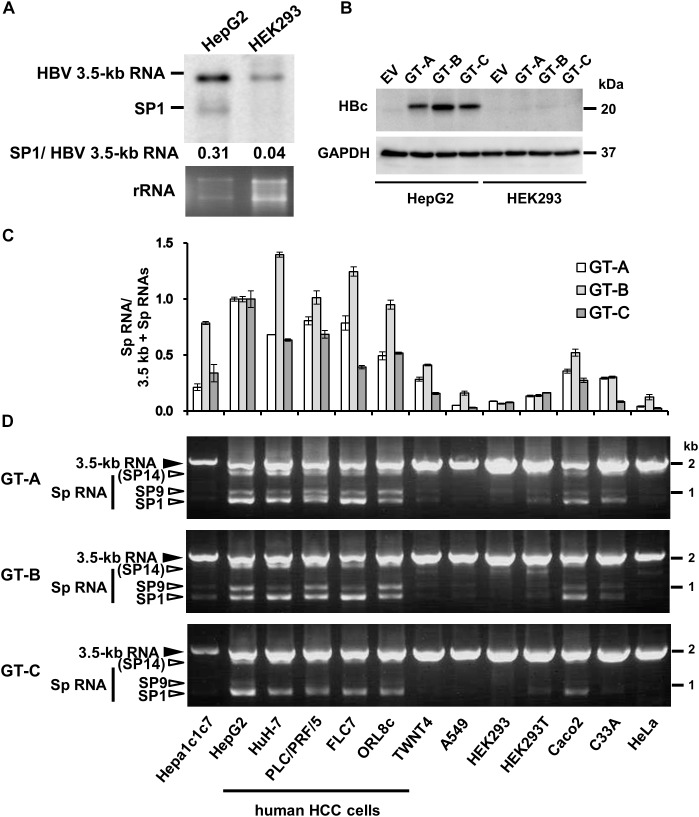
Cell-type dependency of HBV 3.5 kb RNA splicing. **(A)** pUC-HB-Ce was transfected into HepG2 and HEK293 cells. Two days after the transfection, total RNA was extracted from cells and separated on an agarose gel. HBV 3.5 kb RNA and spliced RNA(s) were detected by northern blotting using HBV RNA probe (nt 1998–2447) (Supplementary Figure S1A). Band intensities of 3.5 kb- and SP1 RNAs on the blot were determined by ImageJ software and the ratios of SP1 RNA to 3.5 kb RNA calculated were indicated. Ethidium bromide-stained agarose gel electrophoresis of 18S ribosomal RNA (rRNA) was also shown (bottom). **(B)** HepG2 and HEK293 cells transfected with pUC-HB-Ae (GT-A), -Bj56 (GT-B), -Ce (GT-C) and an empty vector (EV) were analyzed by immunoblotting to detect HBcAg and glyceraldehyde-3-phosphate dehydrogenase (GAPDH). **(C)** qRT-PCR analysis was performed to determine the levels of 3.5 kb RNA and spliced RNA in 13 kinds of cells transfected with pUC-HB-Ae (GT-A), -Bj56 (GT-B), and -Ce (GT-C). The quantity ratios of the spliced RNAs to total 3.5 kb RNA derived RNA species were calculated and those in HepG2 cells were set to 1. Values shown represent means ± SD obtained from three independent samples. **(D)** Total RNAs obtained from the cell samples as shown in **(C)** were used with semi-quantitative RT-PCR. cDNA bands corresponding to unspliced 3.5 kb RNA and its spliced forms (Sp RNA) were detected by agarose gel electrophoresis. Unspliced 3.5 kb RNA, SP9, and SP1 were identified by nucleotide sequencing. SP14 was estimated from the size of PCR product obtained. Cell lines used were indicated at the bottom.

The ratio of the spliced HBV RNAs to unspliced 3.5-kb RNA or total (spliced and unspliced) RNAs derived from 3.5 kb RNA was determined in two ways; based on quantitative- (q) and semi-quantitative RT-PCRs. In experiments with the 1.24-fold full-length HBV genomes but not with their deletion mutants as shown in Figure 1C, 5 and Supplementary Figures S5, S6, spliced forms and total (unspliced plus spliced) RNAs derived from 3.5 kb RNA were separately determined by qRT-PCR as described previously (Sun et al., 2017). In brief, total RNAs were extracted from transfected cells with TRI Reagent (Molecular Research Center, Cincinnati, OH, United States). After treatment with inhibitors for DNase I and RNase, cDNA templates were synthesized and were quantified by qPCR using the SYBR qPCR Mix kit (Toyobo, Osaka, Japan) with the primer sets, unSpF; 5′-TCCCTCGCCTCGCAGACG-3′ and unSpR; 5′-GTTTCCCACCTTATGAGTC-3′ for unspliced 3.5 kb RNA, and SpF; 5′-CCGCGTCGCAGAAGATCT-3′ and SpR; 5′-CTGAGGCCCACTCCCATAGG-3′ for 3.5 kb-derived spliced RNAs. Data obtained were used for calculating the ratio of the spliced RNAs to total (unspliced plus spliced) RNAs derived from 3.5-kb RNA.

In experiments containing both with wild-type and mutated HBV genomes as shown in Figure 3, 4, semi-quantitative RT-PCR was used because of difficulty in designing primers/probe for qRT-PCR that allows proper quantification of HBV RNA isoforms generated from a series of deletion mutants. cDNA templates were prepared as described above and HBV cDNAs derived from spliced- and unspliced 3.5 kb RNAs were amplified by PCR with the primer sets, pgRNAF; 5′-AGCCTCCAAGCTGTGCCTTGGGTG-3′ and pgRNAR; 5′-AACCACTGAACAAATGGCACTAGTAAACTGAGC-3′. PCR products were visualized by agarose gel electrophoresis. Image intensities corresponding to each spliced form as well as an unspliced form were quantified using the image processing program ImageJ (Schneider et al., 2012), followed by calculating the quantity ratio of the spliced RNAs to the unspliced RNA. A schematic diagram of positions of PCR primers used is indicated (Supplementary Figure S1A).

### Statistical Analysis

The significant statistical difference of HBV pgRNA splicing ratio between cell-types was determined by Wilcoxon rank sum test. For other parametric measures, significance between two groups was determined using Student’s *t*-test, while significance between four or more groups was determined using one-way ANOVA with Dunnett’s test. *p*-values < 0.05 were considered statistically significant.

## Results

### Cell-Type Dependency of HBV 3.5 kb RNA Splicing

We first compared expression of HBV 3.5 kb, pregenomic plus precore RNA and spliced form(s) derived from the 3.5 kb RNA in human liver-derived and non-liver-derived cells that were cultured for 2 days after transfection with the 1.24-fold HBV genome. Northern blotting with pregenome/precore probe showed that two bands corresponding to unspliced 3.5 kb RNA and 3.5 kb RNA -derived, 2.2 kb spliced RNA (SP1) lacking ∼1.2 kb intron, respectively, were more abundant in HepG2 cells compared to HEK293 cells (Figure 1A). Higher expression of 3.5 kb RNA and HBcAg in HepG2 compared to those in HEK293 cells were confirmed by qRT-PCR (Supplementary Figure S1B) and Western blotting (Figure 1B), respectively. It is noted that HepG2 and HEK293 cells exhibited similar transfection efficiency as monitoring expression of fluorescencent protein in the transfected cells (Supplementary Figure S1C). From quantification of the band intensity in northern blotting data, the quantity ratio of SP1/unspliced 3.5 kb RNAs appears to be higher in HepG2 cells than that in HEK293 cells (Figure 1A).

To analyze possible cell-type dependency of HBV 3.5 kb RNA splicing, a panel of cell lines derived from human and mouse hepatic- as well as human non-hepatic tissues was used for expression of the HBV genome derived from GT-A, -B, and -C. Both quantitative (Figure 1C) and semi-quantitative (Figure 1D) RT-PCR analyses showed variation of the splicing efficiency among cell lines tested. It is of interest that the ratio of spliced/unspliced 3.5 kb RNAs was higher in all five kinds of cell lines derived from human HCC (HepG2, HuH-7, PLC/PRF/5, FLC7, and ORL8c), compared to other cell types; mouse hepatoma (Hepa1c1c7), human hepatic stellate cell derived (TWNT4), human non-hepatic cells (A549, HEK293, HEK293T, Caco2, C33A and HeLa). To ask whether such a cell-type dependent manner of the splicing was also observed in the splicing of another viral RNA, a construct carrying the SV40 late gene (SV40-L WT) was introduced into HepG2, HuH-7, PLC/PRF/5, A549, HeLa and HEK293 cells and the RNA expression was analyzed by RT-PCR. Spliced RNAs including the dominant form that corresponds to the SV40 VP1 RNA were similarly detectable both in hepatic and non-hepatic cells (Supplementary Figure S3; upper). It is thus likely that the splicing efficiency of HBV 3.5 kb RNA is dependent on cellular context and the RNA is preferentially spliced in cells derived from human liver; in particular, HCC.

### Identification of *Cis*-Regulatory Elements Involved in HBV 3.5 kb RNA Splicing in Cell-Type Dependent and - Independent Manners

**FIGURE 2:**
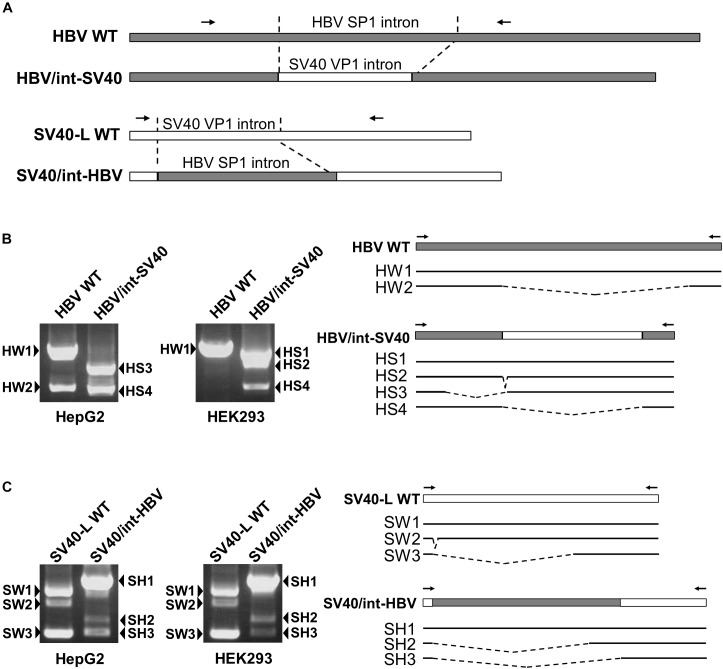
Identification of *cis*-regulatory elements involved in HBV 3.5-kb RNA splicing in cell-type dependent and -independent manners. **(A)** A schematic representation of constructs and PCR primers used in the intron-swapping experiment is indicated. **(B)** HBV WT and HBV/int-SV40 were expressed in HepG2 and HEK293 cells. PCR products were analyzed by agarose gel electrophoresis (left). Schematic of unspliced and spliced forms detected is shown (right). **(C)** SV40-L WT and SV40/int-HBV were expressed in the cells and PCR products were analyzed as shown in **(B)**.

To identify regulatory *cis*-element(s) involved in controlling HBV 3.5 kb RNA splicing in the cell-type dependent manner, two swapped-region constructs (HBV/int-SV40 and SV40/int-HBV) in which the HBV sequence corresponding to the intron for majority of the HBV spliced RNAs and the SV40 sequence of the intron for the viral late gene were swapped in the plasmids that contain the SV40 late gene (SV40-L WT) and the1.24-fold HBV genome derived from GT-C (HBV WT), respectively, were generated (Figure 2A) and the splicing patterns expressed from the swapped constructs were tested in HepG2 and HEK293 cells (Figure 2B,C). In HepG2 cells expressing HBV/int-SV40, two spliced RNAs termed HS3 and HS4 but not unspliced RNA were observed. In HEK293 cells, two spliced RNAs; HS2 and HS4 in addition to unspliced RNA (HS1) were detectable (Figure 2B). By contrast, the RNA pattern expressed from SV40/int-HBV was comparable in HepG2 and HEK293 cells. In both cells, the ratio of spliced/unspliced RNAs appeared to be lower in case of SV40/int-HBV compared to SV40-L WT (Figure 2C), suggesting the existence of splicing silencer(s) in the intron region of HBV 3.5 kb RNA which presumably functions in the cell-type independent manner. To confirm this, SV40/int-HBV was further expressed in HuH-7, PLC/PRF/5, A549 and HeLa as well as HepG2 and HEK293 cells. As expected, expressed RNAs from SV40/int-HBV were similarly processed in all cell lines tested in contrast to the RNAs from HBV/int-SV40 and their splicing efficiency was lower compared to that from SV40-L WT (Supplementary Figure S3; upper, middle and lower).

Regarding the role of HBV exonic region in the splicing regulation, while a certain level of unspliced SV40 RNA in addition to the spliced forms was detectable in SV40-L WT-expressing HepG2 cells (Figure 2C), most of all RNAs expressed from HBV/int-SV40 (Figure 2B) were spliced in the cells. By contrast, higher splicing efficiency caused by HBV/int-SV40 was not observed in HEK293 cells; it appears that the ratio of spliced/unspliced RNAs was lower in the cells expressing HBV/int-SV40 (Figure 2B) compared to SV40-L WT (Figure 2C). Such a difference in splicing efficiency which is higher in HBV/int-SV40 expression compared to SV40-L WT was also observed in HuH-7 and PLC/PRF/5 but not in A549 and HeLa cells (Supplementary Figure S3; upper, middle, and lower). It is thus likely that the sequence(s) within the exon region of majority of the HBV spliced RNAs plays a positive role in its splicing regulation in the cell-type dependent manner.

### Characterization of the Intronic Sequence Important for Silencing of the 3.5-kb RNA Splicing Within the Viral Major Intron Region

**FIGURE 3:**
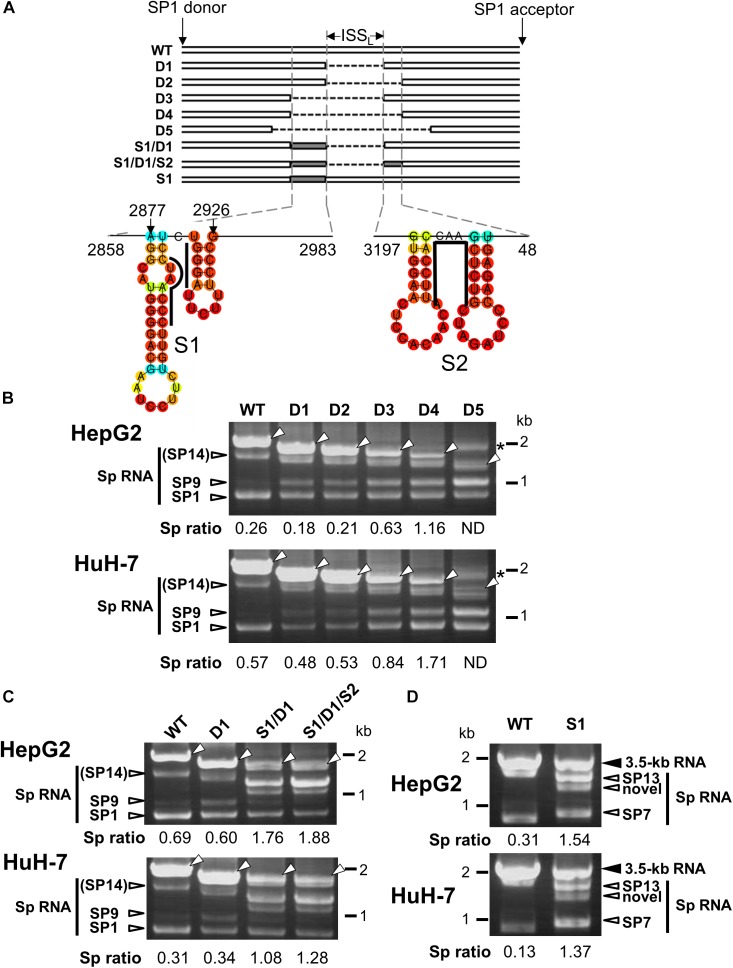
Characterization of the intronic sequence important for silencing of the 3.5 kb RNA splicing. **(A)** A schematic representation of mutated HBV genomes derived from GT-C with deletions and/or substitutions within the major intron region is shown. Putative secondary structures predicted by the CentroidFold (http://rtools.cbrc.jp/centroidfold/) in nt 2858–2983 and 3197-48 regions are indicated. Each predicted base pair is colored with the heat color gradation from blue to red corresponding to the base-pairing probability. Bold lines are drawn along the sequences targeting to introduce substitution mutations S1 and S2. **(B)** RT-PCR analysis of HBV RNAs expressed from WT and a series of deletion-mutated HBV genomes (D1–D5) in HepG2 and HuH-7 cells. The quantity ratio of the spliced RNAs to the unspliced RNA (Sp ratio) was determined. ND, not determined. Open arrows, unspliced forms. ^∗^cDNA products with aberrant sizes. **(C)** As described in **(B)** but HBV mutated genomes S1/D1 and S1/D1/S2 in addition to WT and D1 were used for RNA expression. **(D)** As in **(C)** but S1 mutant and WT were expressed in the cells. Bands corresponding to unspliced 3.5 kb RNA and its spliced forms (Sp RNA) were indicated.

A previous study has identified an ISS_L_ for HBV pregenomic RNA splicing in expression of HBV minigene constructs under the control of heterologous CMV-IE promoter (Chowdhury et al., 2011). Since we also found that the HBV major intron region contains a *cis*-regulatory element that silences the usage of adjacent splice sites of the 3.5 kb RNA in the context with the viral genome replication as shown above, a series of the 1.24-fold HBV genome constructs derived from GT-C with partial deletions corresponding to the intronic sequence were generated to identify the region that contributes to negative regulation of the splicing (Figure 3A). Their splicing silencer activity was analyzed by comparing processing patterns of expressed RNAs in HepG2 and HuH-7 cells (Figure 3B). Either deletion mutant D1 or D2, in which ISS_L_ or nt 2984-48 region was deleted, respectively, has a moderate influence on the ratio of spliced/unspliced RNAs. Expression of D3 mutant resulted in a marked increase in the ratio and D4 displayed the highest splicing efficiency. Further deletion(s) such as D5 caused generation of some aberrant RNA product(s) whose size(s) are larger than the expected unspliced form.

The possible contribution of the regions spanning nt 2858–2983 and 3197-48 which potentially contain several stem-loop structures (Figure 3A) to the splicing regulation was further tested by introducing substitution mutations in the respective sequences of D1 mutant to disrupt the base-pairs of putative stem-loops, leading to changing the secondary structures of the region. Expression of both mutants; S1/D1 and S1/D1/S2 resulted in generation of novel spliced RNA species and increasing significantly the ratio of spliced/unspliced RNAs (Figure 3C). To pinpoint the sequence response for the novel intronic splicing silencer, a mutated genome S1 with substitutions at nt 2902–2916 region (Figure 3A) was assessed. Expression of S1 led to a decreased level of unspliced RNA and change in the spliced RNA pattern (Figure 3D), indicating reduction of the splicing silencer activity by the substitution mutation. Nucleotide sequencing showed that previously reported spliced RNAs; SP7 and SP13 (Bayliss et al., 2013) and a novel spliced RNA (Supplementary Figure S4) were detectable in cells expressing S1 mutant genome.

**FIGURE 4:**
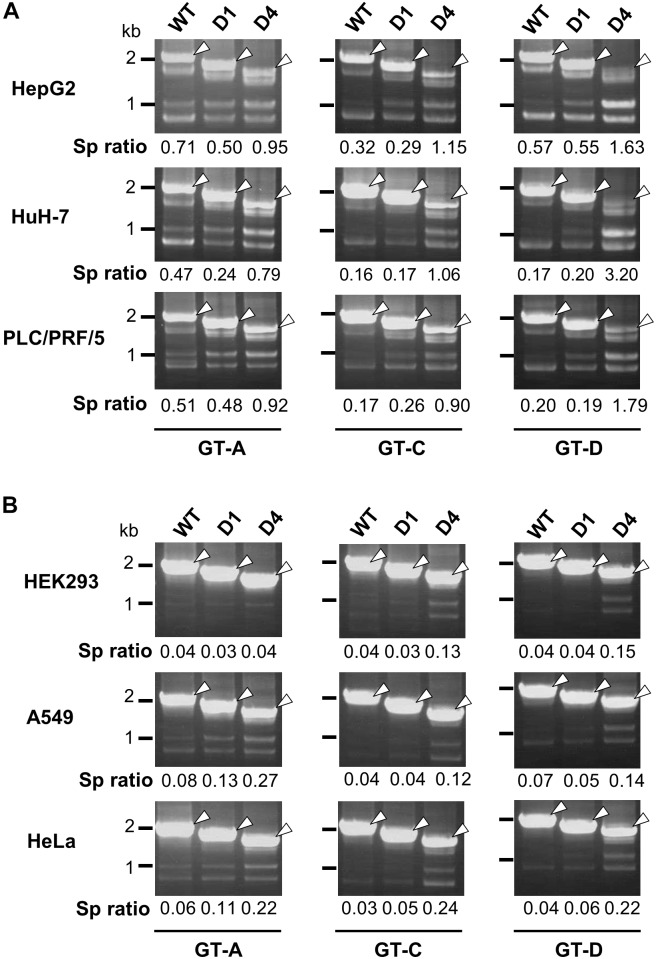
Effect of deletions in intron region of the HBV genome on the splicing efficiency in human HCC and non-hepatic cells. RT-PCR analyses of HBV RNAs expressed from WT and deletion-mutants corresponding to D1 and D4 derived from GT-A (deleted regions; nt 2990–3202 in D1, nt 2864-48 in D4), -C (nt 2984–3196 in D1, nt 2858-48 in D4) and -D (nt 2951–3163 in D1, nt 2858-48 in D4) in human HCC (HepG2, HuH-7 and PLC/PRF/5) cells **(A)** and in non-hepatic (HEK293, A549, and HeLa) cells **(B)**. Sp ratio; the quantity ratio of the spliced RNAs to the unspliced RNA. Open arrows, unspliced forms.

In nt 2858–2983 region, two stem-loop structures that are important for the silencing activity are predicted to exist in the sequence spanning nt 2877–2926 (Figure 3A). Collectively, we concluded that the intron region of nt 2877–2926, located upstream from the previously reported ISS_L_, functions as a potent splicing silencer and is termed as ISS_UL_ (ISS upstream from ISS_L_). Further experiments with the mutated genome D4 showed that higher splicing ratios in cells expressing D4 compared to expression of D1 and wild-type genomes were observed in any cell lines as well as HBV genotypes tested. The results suggested that the sequence containing ISS_UL_ and ISS_L_ in the major intron region of 3.5 kb RNA displayed the splicing silencing activity independent of HBV GTs and of cell backgrounds (Figure 4A,B).

### Characterization of Genotype/Strain-Dependent Variation in the Efficiency of the 3.5-kb RNA Splicing

**FIGURE 5:**
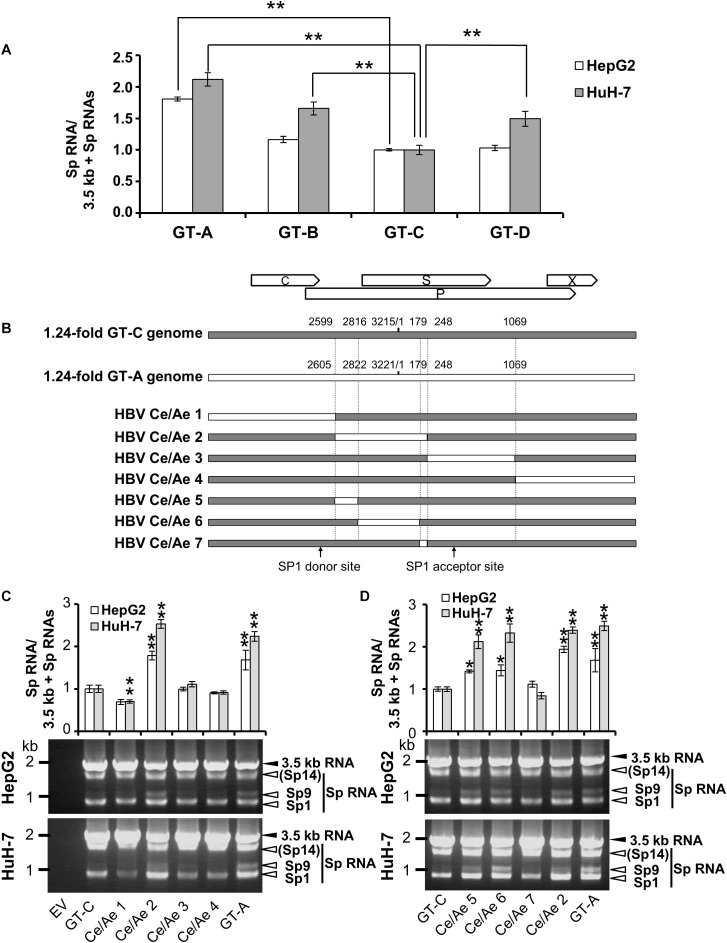
Diversity in the splicing efficiency among HBV genotypes/strains. **(A)** pUC-HB-Ae (GT-A), -Bj56 (GT-B), -Ce (GT-C) or -D_Ind60 (GT-D) was transfected into HepG2 and HuH-7 cells. Two days after the transfection, total RNA was extracted from cells and analyzed by qRT-PCR to determine the levels of 3.5 kb RNA and spliced RNAs. The quantity ratios of the spliced RNAs to total 3.5 kb RNA derived RNA species were calculated and that of GT-C was set to 1. **(B)** A schematic representation of a series of mutated HBV genomes replacing parts of the GT-C sequence with corresponding parts of GT-A sequence. Positions of donor and acceptor sites of SP1 RNA are indicated. **(C)** qRT-PCR analysis was performed to determine 3.5 kb RNA and spliced RNA levels in HepG2 and HuH-7 cells transfected with pUC-HB- Ce (GT-C), -Ae (GT-A), HBV Ce/Ae1, /Ae2, /Ae3 or /Ae4. The quantity ratios of the spliced RNAs to total 3.5 kb RNA derived RNA species were calculated and those in GT-C-expressing cells were set to 1 (upper). Representative pattern for RT-PCR result indicating expression of unspliced and spliced (Sp) RNAs derived from 3.5 kb RNA in the transfected HepG2 (middle) and HuH-7 cells (lower). **(D)** As in **(C)** but mutated genomes HBV Ce/Ae5, /Ae6, /Ae7 as well as GT-C, -A and HBV Ce/Ae2 were used for expression of HBV RNAs. Values represent mean ± SD (HepG2; *n* = 2, HuH-7; *n* = 3). ^∗^*p* < 0.05, ^∗∗^*p* < 0.01, by one-way ANOVA followed by Dunnett’s test compared to GT-C.

As a characterization of splicing regulation of HBV RNAs, we next asked how splicing efficiency of 3.5 kb RNA varies among HBV genotypes or strains. Four kinds of HBV genomes derived from GT-A, -B, -C, and -D were expressed in HepG2 and HuH-7 cells and the ratios of spliced/unspliced RNAs were compared at 2 days post-transfection (Figure 5A). Among HBV GTs/strains tested, the splicing efficiency of the 3.5 kb RNA expressed from the GT-A clone and that from the GT-C clone was highest and lowest, respectively, in both cells. Similar results were obtained when the splicing efficiency was determined at 4 days post-transfection (Supplementary Figure S5). To identify the viral sequence that has potentially influence on GT/strain-dependent variation in the splicing efficiency, parts of HBV sequence from the GT-C clone were serially replaced by the corresponding parts from the GT-A clone in the genome (Figure 5B) and expressed. The 3.5 kb RNA expressed from the chimeric clone Ce/Ae2 that carries GT-A-derived nt 2605-248 sequence in the GT-C basis was efficiently spliced comparable to that from the wild-type GT-A clone (Figure 5C). The 3.5 kb-RNA splicing from chimeric mutants Ce/Ae5-Ce/Ae7 was further assessed. Splicing efficiency of the viral RNAs derived from Ce/Ae5 and Ce/Ae6 but not from Ce/Ae7 was increased compared to that from wild-type GT-C clone (Figure 5D). For the region-swapping experiment, a chimeric clone Ae/Ce that is GT-A based counterparts of Ce/Ae2 was constructed (Supplementary Figure S6A). As expected, the 3.5 kb RNA expressed from Ae/Ce were less efficiently spliced compared to the wild-type GT-A clone and the splicing efficiency of Ae/Ce-derived RNA was as low as that of the wild-type GT-C clone (Supplementary Figure S6B). Although evidence obtained from the GT-A and -C clones in this study was limited, it may be likely that a considerable part (795 nt) within the major intron region is possibly involved in GT/strain-dependent variation in the efficiency of the 3.5-kb RNA splicing.

## Discussion

It is generally considered that alternative RNA splicing is essential for the life cycles of nuclear-replicating viruses because their post-transcriptional processing is one of the major mechanisms in increasing the diversity of proteins translated from a limited number of viral genes required to initiate and maintain the viral proliferation. However, knowledge of a cell-type dependent manner of the viral RNA splicing and its regulatory mechanism is quite limited (Ge and Manley, 1990; Butera et al., 1994). In this study, through analysis with 13 kinds of cell lines, we found cell-type dependency of splicing of HBV 3.5 kb RNA. The viral RNA is relatively efficiently spliced in human HCC cells but not in cells derived from human hepatic stellate, mouse hepatoma and human non-hepatic cells (Figure 1C). It is possible that difference in transcription efficiency of the HBV genome may have an influence on the splicing regulation. In fact, expression levels of HBV RNAs in human HCC-derived, HepG2 and HuH-7 cells were highest among cell lines tested. However, the HBV RNA levels expressed in other human HCC-derived, PLC/PRF/5, FLC7 and ORL8c cells where the viral RNAs can be efficiently spliced like HepG2 and HuH-7 cells were quite low. The expression levels in PLC/PRF/5, FLC7 and ORL8c cells were lower compared to those in some non-HCC cells such as HEK293 and HEK293T cells. Thus, it is likely that cell-type dependency of HBV splicing efficiency cannot be explained only by expression level of the viral RNAs. These findings may suggest that RNA splicing is a key determinant for optimizing regulation of HBV life cycle and the host range. HBV RNA splicing is a common event during chronic infection (Sommer and Heise, 2008). Several singly and multiply spliced isoforms derived from the HBV 3.5 kb RNA have been identified in liver tissues and sera obtained from HBV-infected individuals as well as tissues and cultured cells of HBV-replicating models such as transgenic mice and transfected cells (Sommer and Heise, 2008). Although the spliced variants derived from 3.5 kb RNA are basically unable to replicate autonomously because of no production of functional polymerase, it may be possible to replicate in the presence of unspliced RNA intermediate, the pregenomic RNA. The most frequently detectable HBV spliced form SP1, which is generated through the removal of a ∼1.2 kb intron from the 3.5 kb RNA (Su et al., 1989; Suzuki et al., 1989). SP1 was reported to encode an hepatitis B splice-generated protein HBSP, which contains a small portion of the N-terminal viral polymerase fused with a new open-reading frame produced by the splicing (Soussan et al., 2000). In addition, SP1 has the potential to encode HBcAg with the C-terminal cysteine deleted. A doubly spliced form, SP10, has also been characterized and reported to encode ∼15 kDa hepatitis B doubly spliced protein (Chen et al., 1989). Although possible implication of these proteins encoded by spliced RNAs in HBV replication has been suggested (Ma et al., 2009; Bayliss et al., 2013), further studies are required to explore and evaluate the biological significance of the spliced RNAs and proteins encoded by the RNAs in HBV replicative life cycle.

In general, sequence features/elements outside of the canonical splice site and branch site sequence located near the 5′ and 3′ ends of introns can be involved in recognizing authentic splice sites by the splicing machinery among a lot of sequences that match the consensus motifs. These elements are conventionally classified as exonic splicing enhancers, exonic splicing silencers, intronic splicing enhancers and ISSs. As *cis*-regulatory elements for splicing of HBV 3.5 kb RNA, the PRE located at nt 1217–1582 (Heise et al., 2006) and the ISS_L_ located at 2951–3163 (nucleotide numbering according to AY945307) (Chowdhury et al., 2011) were identified using constructs under the control by the heterologous promoters. In this study, given the results showing the difference in cell-type dependency of the splicing efficiency between HBV and SV40 (Figure 1C and Supplementary Figure S2), intron-swapping experiments were performed (Figure 2). The results suggest that the certain sequence(s) within the exon region of majority of the HBV spliced RNAs positively regulate the splicing in the cell-type dependent manner. From further analyses with deletion and substitution mutations within the major HBV intron region, we identified a novel element ISS_UL_ that functions as ISS located at nt 2877–2926 region, upstream of ISS_L_. It appears that the splicing silencing activity of ISS_UL_ is more potent compared to that of ISS_L_ (Figure 3C,D) and that the intron region containing ISS_UL_ and ISS_L_ functions effectively independent of the viral GTs/strains and of cell backgrounds (Figure 4). By comparing data between hepatic (Figure 4A) and non-hepatic (Figure 4B) cells, an increase in the ratio of spliced to unspliced products by deletion with nt 2864-48 region in non-hepatic cells seems to be less evident than that in hepatic cells. One reason may be that, in addition to ISS_UL_ and ISS_L_, a putative positive regulator in the exon region, as mentioned above, is potentially involved in controlling the HBV splicing particularly in hepatic cells. The significance of RNA secondary structures in molecular mechanisms including alternative splicing is well described. It is thus important to consider that structural conformation is a key to the element for splicing regulation. Interestingly, the ISS_UL_ is predicted to contain two stem-loop structures in close proximity (Figure 3A). Database analyses, together with the secondary structure prediction, demonstrated that nucleotide sequence in the ISS_UL_ region is largely well conserved and in particular the second stem-loop structure is completely conserved among GT-A-D clones.

It is known that most of all corresponding donor and acceptor sites for major splicing derived from the 3.5 kb RNA are correctly identified in consensus sequences of the viral GTs A-D (Candotti and Allain, 2017). In contrast, several studies with clinical samples reported that efficiency of the 3.5 kb RNA splicing and nature of splice variants differ from patient to patient ranging from undetectable splicing to extensive and genotype/viral strain-dependent splicing (Wu et al., 1991; Gunther et al., 1997; Sommer et al., 2000; El Chaar et al., 2012).

In this study, the ratio of spliced to unspliced 3.5 kb RNA expressed from four kinds of HBV clones individually derived from GTs A-D in HepG2 and HuH-7 cells was compared. The ratios obtained from GT-A and GT-C clones tended to be highest and lowest, respectively. Further findings from the region-swapping experiments suggest that ∼two-third part of the major intron region may be participated in genotype/strain-dependent variation in the splicing efficiency. Thus, besides the identified ISS regions that are well conserved among HBV strains, unidentified *cis*-elements that are responsible for positive- or negative splicing regulation and are functional in a GT/strain-dependent fashion may be present within the intron.

Upon infection, HBV hijacks the host splicing machinery to develop regulatory mechanisms for alternative splicing of the viral transcripts. Our finding that the HBV 3.5 kb RNA is efficiently spliced in human hepatic cells among various types of cells may suggest its biological significance as a host range determinant. In contrast, maintenance of an appropriate amount of the unspliced form is indispensable to supplying the replication intermediate during the viral replication cycles. Thus, a crucial aspect for HBV alternative splicing is accurate control of proper ratios of spliced to unspliced forms. A shift in the viral splicing and change in the spliced/unspliced RNA ratio might be achieved by altering the balance of nuclear factors that recognize either splicing enhancers or silencers and mediate the catalytic steps of the splicing reaction. Further studies on mapping putative exonic enhancer(s) responsible for acting in a cell-type dependent manner, identification of host factors that positively and negatively regulate the splicing and elucidation of their regulatory mechanisms are important to better understand HBV RNA regulation and the significance of the viral alternative splicing.

## Author Contributions

NI, KN, SS, and MI performed the experimental work. NI and KN prepared the figures and table. KN and TS contributed in study conception and data interpretation. NI, KN, and TS wrote the manuscript. All authors participated in data interpretation and contributed to manuscript revision.

## Conflict of Interest Statement

The authors declare that the research was conducted in the absence of any commercial or financial relationships that could be construed as a potential conflict of interest.
