# Supplementary material for: Cell Type Diversity in Hepatitis B Virus RNA Splicing and Its Regulation
Source: Front Microbiol. 2019 Feb 8;10:207. doi: 10.3389/fmicb.2019.00207 (PMC6375855; doi:10.3389/fmicb.2019.00207)
Supplement: Supplementary file 1 [file Presentation_1.pdf]

Supplementary Material

Cell type diversity in hepatitis B virus RNA splicing and its regulation

Noriomi Ito, Kenji Nakashima, Suofeng Sun, Masahiko Ito, and Tetsuro Suzuki*

*Correspondence: Tetsuro Suzuki; tesuzuki@hama-med.ac.jp

Supplementary Table and Figures

	2877 … 2926
WT	AGGCATGGGGACGAATCCTTCTGTTCCCAATCCTCTGGGATTCTTTCCCG
S1	AGGCATGGGGACGAATCCTTCTGTTGGGTTAGCTCTCCCTTTCTTTCCCG

	3197 … 48
WT	GTGGAACTCCACAACATTCCACCAAGCTCTGCTAGATCCCAGAGT
S2	GTGGAACTCCACAACAAAGGTGGTTCGAGACCTAGATCCCAGAGT

Supplementary Table S1. Nucleotide sequences of the regions introducing substitution mutations S1 and S2 (Figure 3A) in pUC HBV-Ce-S1/D1, -S1/D1/S2 and –S1 were shown. Mutated nucleotides within S1 and S2 and the corresponding parts of wild-type (WT) sequences are underlined. Nucleotide numbering was according to the consensus sequence of HBV genotype C.

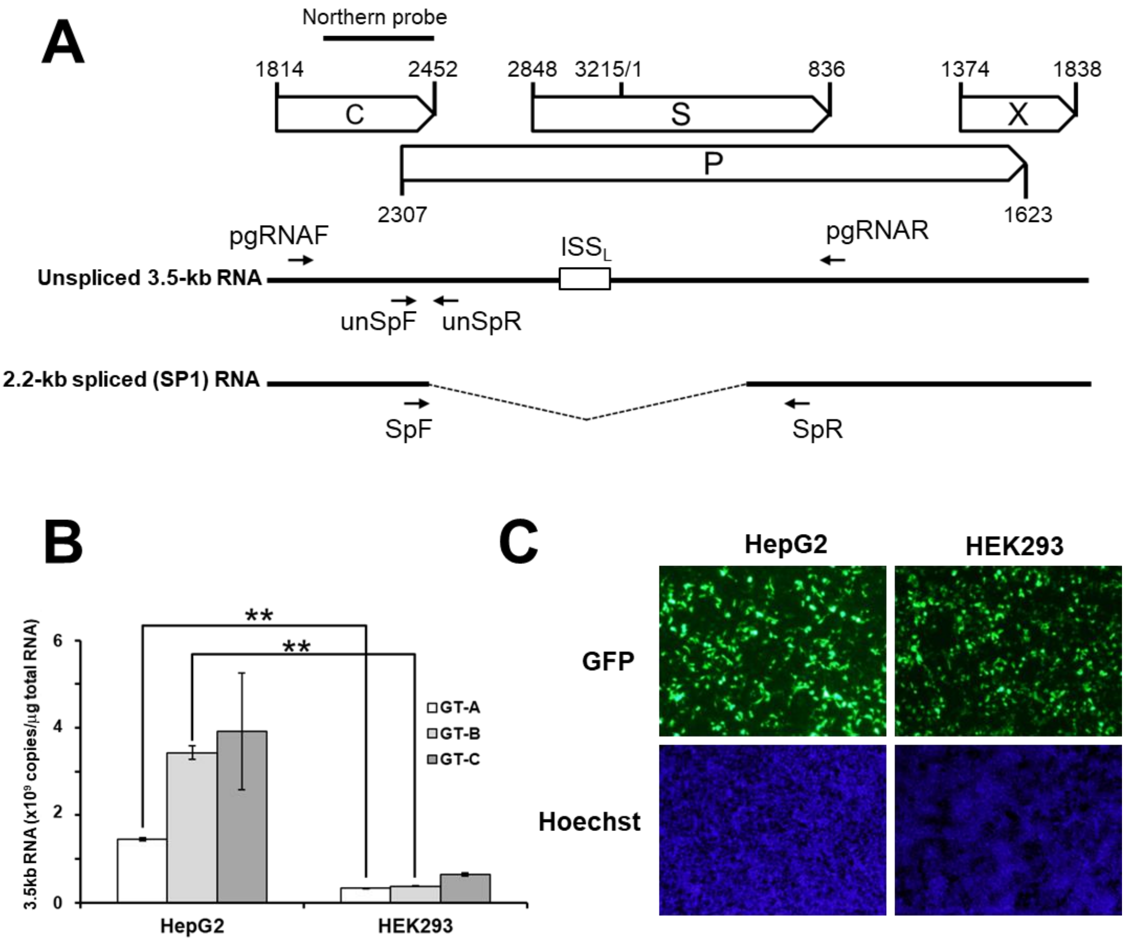

Supplementary Figure S1. (A) A schematic diagram of 3.5 kb- and SP1 RNAs. Locations of RNA probe for northern blotting, PCR primers used in this study and intronic splicing silencer-long (ISS_L) region are indicated (numbering according to GT-C). (B) Expression of unspliced 3.5 kb RNA in HepG2 and HEK293 cells. Two days after transfection with pUC-HB-Ae (GT-A), -Bj56 (GT-B) and -Ce (GT-C), the cells were subjected to qRT-PCR. Values represent the means ± SD (n=2). Student *t* test; $**p<0.01$. (C) Comparison of transfection efficiency in HepG2 and HEK293 cells. Transfection of the cells with a GFP-expression plasmid, pmaxGFP (Lonza, Basel, Switzerland), was performed, followed by immunofluorescence analysis. Nuclei were stained with Hoechst 33258.

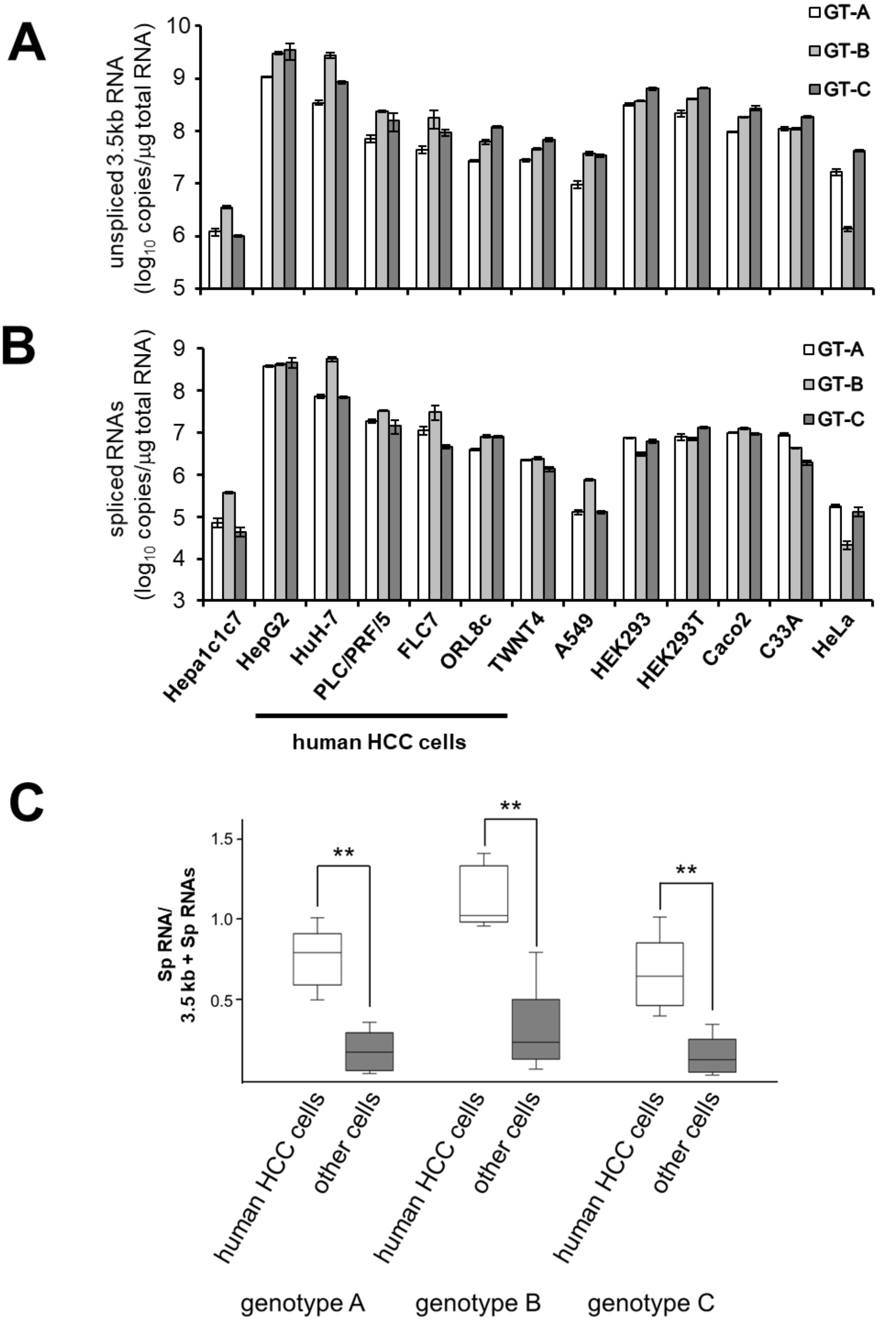

Supplementary Figure S2. Cell-type dependency of HBV 3.5 kb RNA splicing. qRT-PCR analysis was performed to determine the RNA copies of unspliced 3.5 kb RNA (A) and spliced RNAs (B) in 13 kinds of cells transfected with pUC-HB-Ae (GT-A), -Bj56 (GT-B) and -Ce (GT-C). Values shown represent means ± SD obtained from two independent samples. (C) Box plots representing the splicing ratio (Sp RNA/unspliced 3.5 kb + Sp RNAs) between human HCC cells and other cells. The relative values of

splicing ratios in each cell line shown in Figure 1C were divided to two groups; human HCC cells and others. The horizontal midlines and the lower and upper edges of the boxes represent the median value, 25th percentile, and 75th percentile, respectively, of the data values. The lower and upper error bars represent the minimum and maximum values, respectively. Wilcoxon rank sum tests were applied to detect significant differences of the splicing ratios between human HCC cells and other cells. **$p < 0.01$.

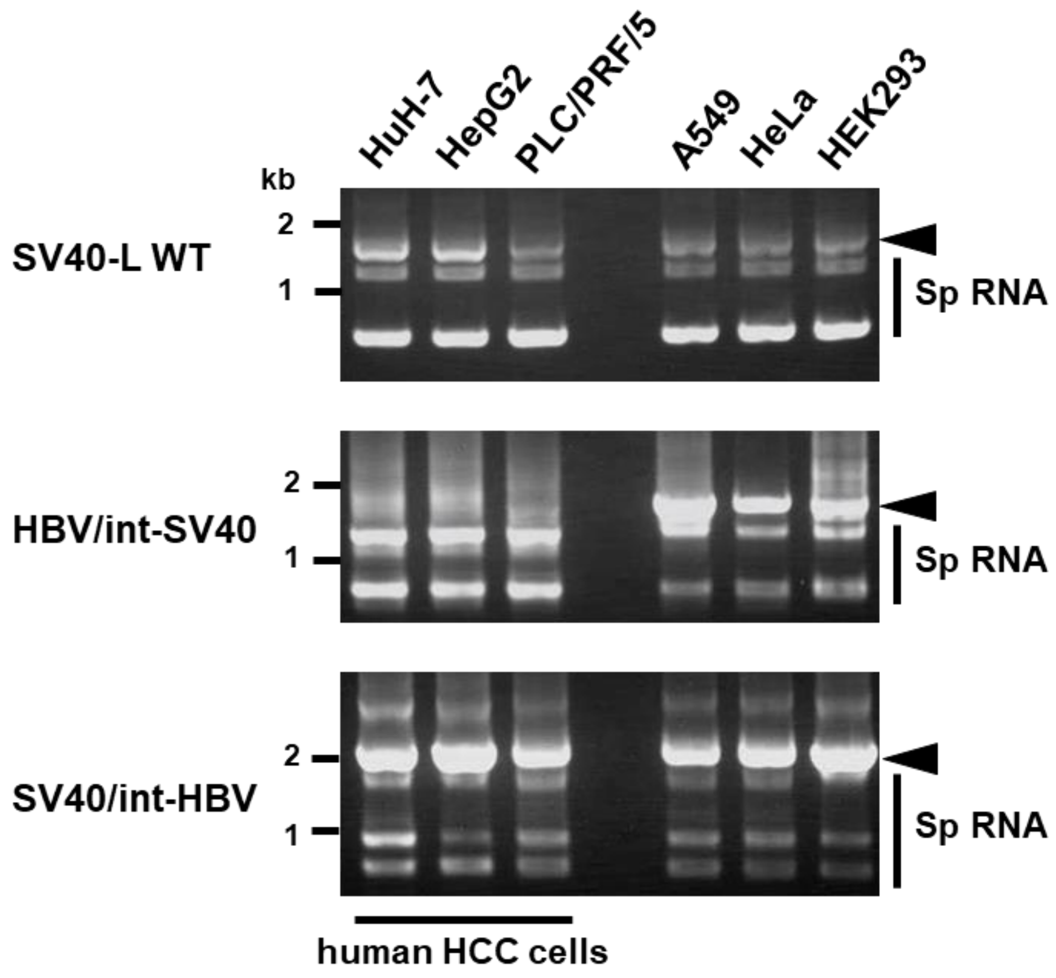

Supplementary Figure S3. pCAG-SV40 (SV40-L WT), pHBV/int-SV40 (HBV/int-SV40) and pSV40/int-HBV (SV40/int-HBV) were expressed in human HCC (HuH-7, HepG2 and PLC/PRF/5) and non-hepatic (A549, HeLa and HEK293) cells. PCR products were analyzed by agarose gel electrophoresis. Bands corresponding to unspliced RNAs are indicated with arrowheads. Spliced forms (Sp RNA) expressed from each construct were shown.

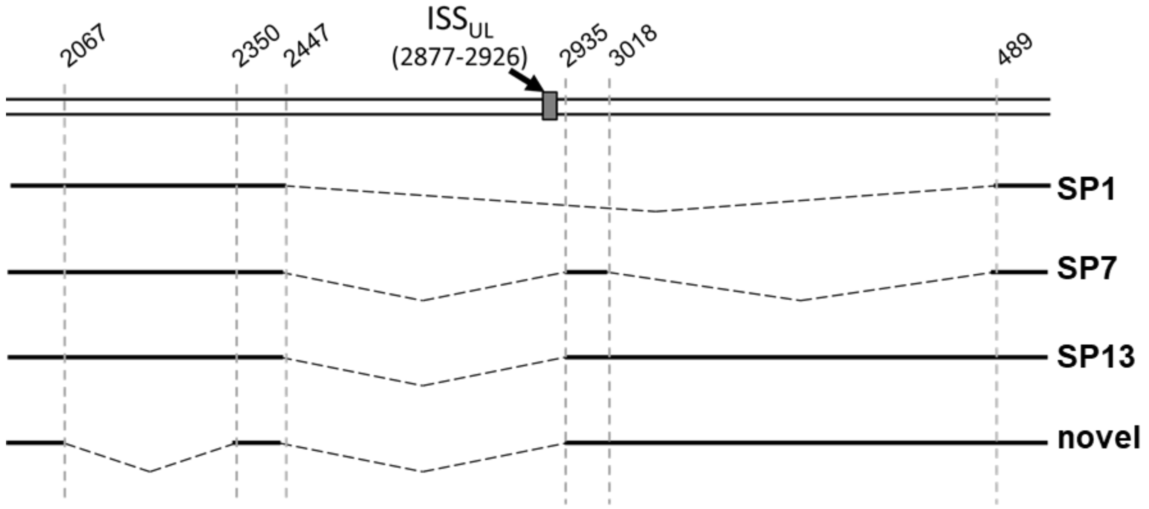

Supplementary Figure S4. Spliced RNAs detected in HepG2 and HuH-7 cells expressed from HBV WT and S1 mutant genomes were mapped. SP1 RNA from WT and SP7, SP13 and novel RNAs from S1 mutant are shown. Position of ISS_{UL} and nt positions for 5' and 3' of exon regions, numbering according to GT-C, are indicated at the top.

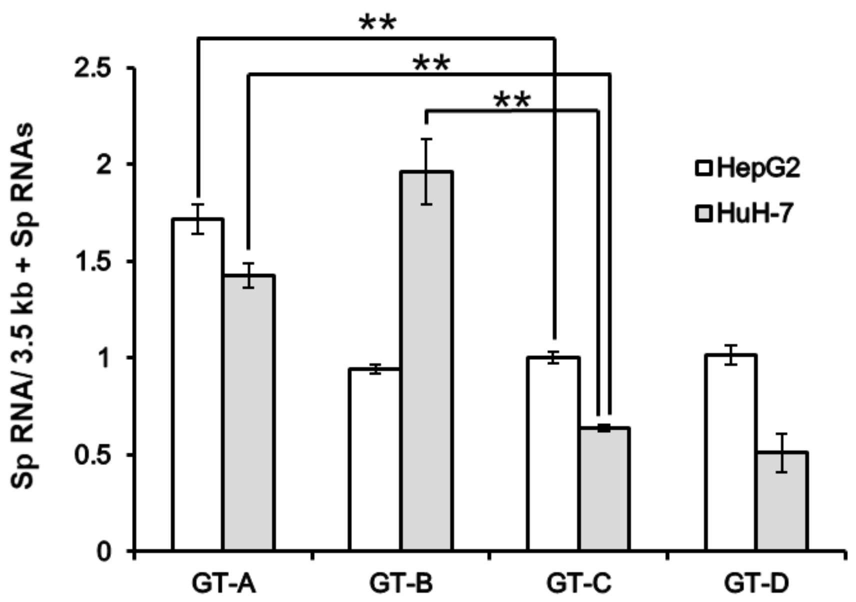

Supplementary Figure S5. The splicing efficiency of HBV 3.5 kb RNA derived from various genotypes was analyzed at 4 days post-transfection. pUC-HB-Ae (GT-A), -Bj56 (GT-B), -Ce (GT-C) or -D_Ind60 (GT-D) was transfected into HepG2 and HuH-7 cells. Four days after the transfection, total RNA was extracted from cells and analyzed by qRT-PCR to determine the levels of 3.5 kb RNA and spliced RNAs. The quantity ratios of the spliced RNAs to total 3.5 kb RNA derived RNA species were calculated and that of GT-C in HepG2 cells was set to 1. Values represent mean ±SD (n=2). **$P < 0.01$, by one way ANOVA followed by Dunnett's test compared to GT-C.

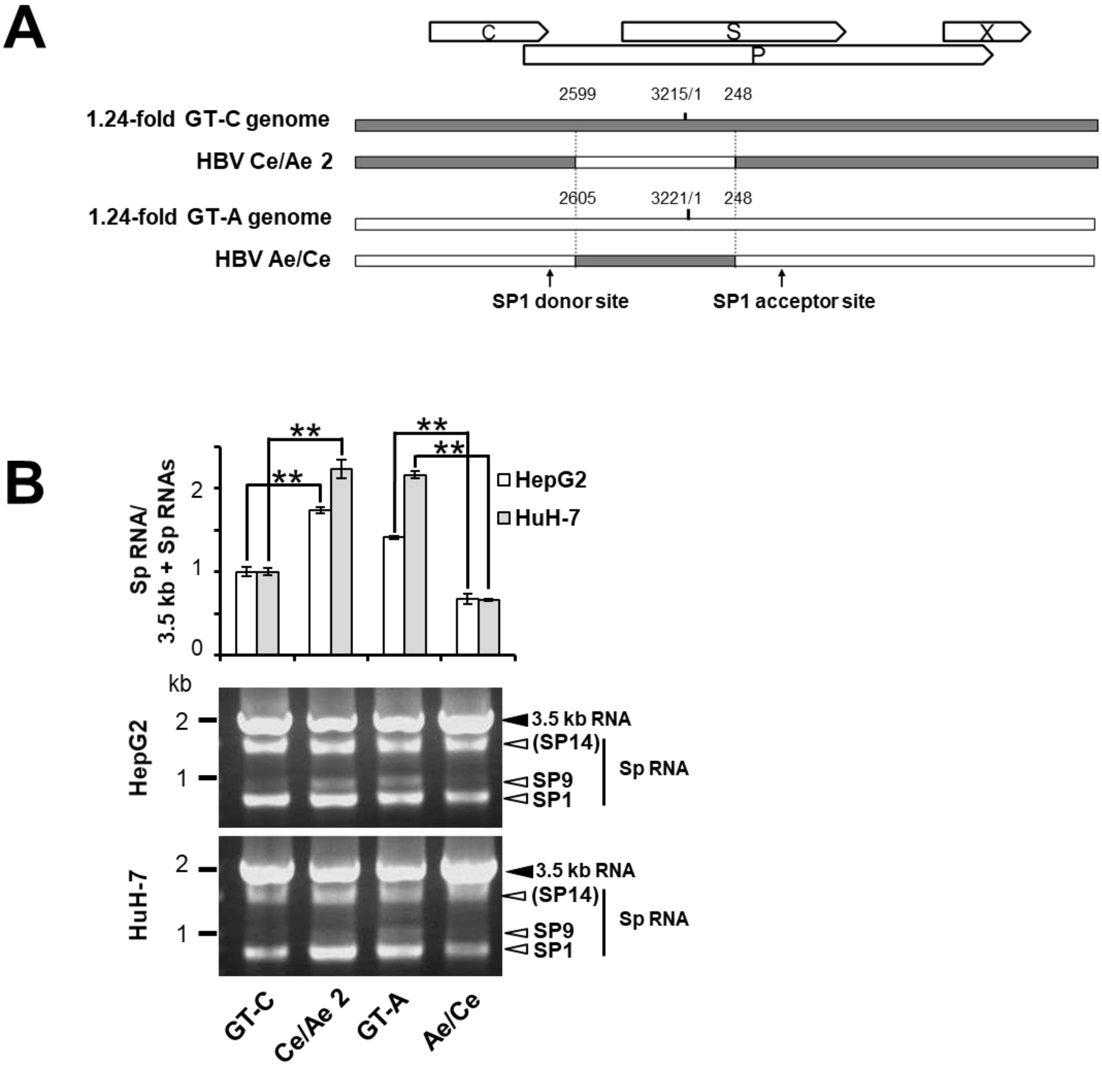

Supplementary Figure S6. (A) A schematic representation of constructs used in the genotype-swapping experiment is indicated. To generate a GT-A-based chimeric plasmid harboring a part of the intron region derived from GT- sequence (HBV Ae/Ce), the nt 2606-247 region in the HBV genome was removed from pUC-HBV-Ae, followed by replacing with the corresponding part from pUC-HBV-Ce. Nucleotide positions based on GT-C and GT-A, respectively, are indicated on the lines for HBV GT-C and GT-A genomes. Positions of donor and acceptor sites for SP1 RNA are shown. (B) qRT-PCR analysis was performed to determine 3.5 kb RNA and spliced RNA levels in HepG2 and HuH-7 cells transfected with pUC-HB- Ce (GT-C), -Ae (GT-A), HBV Ce/Ae2, or Ae/Ce. The quantity ratios of the spliced RNAs to total 3.5 kb RNA derived RNA species were calculated and those in GT-C-expressing cells were set to 1 (upper). Representative pattern for RT-PCR result indicating expression of unspliced and spliced (Sp) RNAs derived from 3.5 kb RNA in HepG2 (middle) and HuH-7 cells (lower) transfected with the plasmids. Values represent the means ± SD (n=2). Student *t* test; ***p*<0.01.
